# Structural brain network topology underpinning ADHD and response to methylphenidate treatment

**DOI:** 10.1038/s41398-021-01278-x

**Published:** 2021-03-02

**Authors:** Kristi R. Griffiths, Taylor A. Braund, Michael R. Kohn, Simon Clarke, Leanne M. Williams, Mayuresh S. Korgaonkar

**Affiliations:** 1 Brain Dynamics Centre, The Westmead Institute for Medical Research, The University of Sydney, Sydney, Australia; 2 Westmead Clinical School, Faculty of Health and Medicine, University of Sydney, Westmead, Australia; 3 Adolescent and Young Adult Medicine, Westmead Hospital, Sydney, Australia; 4 Centre for Research into Adolescents’ Health (CRASH), Sydney, Australia; 5 Psychiatry and Behavioral Sciences, Stanford University, Stanford, CA USA; 6 MIRECC, Palo Alto VA, Palo Alto, CA USA; 7 Discipline of Psychiatry, Sydney Medical School, Westmead, Sydney Australia

**Keywords:** ADHD, Prognostic markers

## Abstract

Behavioural disturbances in attention deficit hyperactivity disorder (ADHD) are thought to be due to dysfunction of spatially distributed, interconnected neural systems. While there is a fast-growing literature on functional dysconnectivity in ADHD, far less is known about the structural architecture underpinning these disturbances and how it may contribute to ADHD symptomology and treatment prognosis. We applied graph theoretical analyses on diffusion MRI tractography data to produce quantitative measures of global network organisation and local efficiency of network nodes. Support vector machines (SVMs) were used for comparison of multivariate graph measures of 37 children and adolescents with ADHD relative to 26 age and gender matched typically developing children (TDC). We also explored associations between graph measures and functionally-relevant outcomes such as symptom severity and prediction of methylphenidate (MPH) treatment response. We found that multivariate patterns of reduced local efficiency, predominantly in subcortical regions (SC), were able to distinguish between ADHD and TDC groups with 76% accuracy. For treatment prognosis, higher global efficiency, higher local efficiency of the right supramarginal gyrus and multivariate patterns of increased local efficiency across multiple networks at baseline also predicted greater symptom reduction after 6 weeks of MPH treatment. Our findings demonstrate that graph measures of structural topology provide valuable diagnostic and prognostic markers of ADHD, which may aid in mechanistic understanding of this complex disorder.

## Introduction

Dysfunction of spatially distributed, interconnected neural systems is thought to be one of the major causes of behavioural disturbances in ADHD^1^. A myriad of studies using functional magnetic resonance imaging (fMRI) have reported altered intrinsic connectivity within and between networks such as the dorsal and ventral attention, salience and default mode networks^2–4^. However, far less is known about the structural networks underpinning these functional disturbances and how they may contribute to ADHD symptomology and prognosis.

Diffusion-weighted imaging (DWI) is a method widely used to examine microstructural brain properties and white matter connections^5^. Numerous studies have reported alterations in microstructural properties within specific tracts in ADHD, predominantly those linking prefrontal, parietal, cerebellar and SC (e.g.^6,7^ see^8^ for a review). These anomalies have also been associated with clinical symptom severity^7^ and the improvement of symptoms across development^9^, demonstrating the relevance of white matter integrity to core symptoms of ADHD.

More recently, our understanding of neural connectivity has been enriched through the application of sophisticated network analysis approaches. These methods allow for the description of complex patterns of neural connectivity (i.e. the ‘connectome’) and quantification of specific network properties^10^. Graph theory is one such method that uses an entirely data-driven approach to produce metrics able to characterise neural networks in terms of information flow and network efficiency. Global efficiency is a quantified measure of how efficiently information can be distributed through the network, with shorter paths (measured by a lower number of network links) representing faster and more efficient transfer of information^11^. Local efficiency of brain regions (or network nodes) essentially unpacks this global efficiency metric by measuring information flow in the vicinity of the node^12^. Most work thus far on brain networks in ADHD has utilised resting-state fMRI (rs-fMRI), which has typically found lower global efficiency in children with ADHD relative to typically developing children (TDC)^13^. White matter architecture is a crucial element underpinning functional activity via the efficient communication between disparate brain regions. There is growing evidence to suggest that structural networks of the brain to some extent shape the processes underlying brain function^14^.

To date, only three studies have used network-based methods in children and adolescents with ADHD using DWI^15–17^. These studies are relatively consistent in their overall network descriptions, finding reduced global efficiency and increased local efficiency in ADHD relative to TDC^15,17^. This corroborates findings from rs-fMRI studies^18^, and indicates reduced communication between more modular, specialised subnetworks in ADHD. Hong and colleagues^16^ identified a network spanning frontal, striatial and cerebellar brain regions that significantly differed in ADHD, with tract integrity in specific regions correlating with attentional deficits. Cao et al.^15^ found increased structural connectivity in orbitofrontal–striatal circuitry and decreased prefrontal dominant circuitry. By contrast, Beare et al.^17^ reported a sub-network of stronger connectivity encompassing bilateral frontostriatal and left occipital, temporal, and parietal regions, with white matter microstructure within these networks being associated with ADHD symptom severity. While there are some inconsistencies in regional findings, collectively, these previous studies suggest there is pathological wiring in white matter networks in ADHD, which may be crucial structural substrates underlying behavioural impairments in ADHD.

Despite previous studies finding links between structural networks and symptoms of ADHD, no previous work has examined whether structural network topology is also linked to prognostic outcomes. MPH is the first-line medication typically used to treat the clinical and cognitive symptoms of ADHD. Its therapeutic effects in ADHD occur via blocking the reuptake of catecholamines, which restores brain activation patterns and functional connectivity towards normative levels, particularly within cortico-striato-cerebellar networks^19–24^. In ~30% of cases, however, it is either ineffective or causes intolerable side-effects^25^. This differential response to medication is inherently linked to individual variation in the neurobiology of the brain^26^. Diffusion imaging measures have successfully predicted pharmacological treatment outcomes in other psychiatric conditions, including major depressive disorder^27^, first episode psychosis^28^ and bipolar disorder^29^. Better characterisation of the biological profile associated with successful MPH therapy will help further our understanding of ADHD aetiology, and may aid in the identification of novel treatment targets for non-responders.

Here, we used diffusion tractography and graph theoretical analyses to compare white matter whole-brain structural networks in children and adolescents with ADHD relative to TDC. Critically, we also examined whether differences in network features were associated with better response to MPH treatment or symptom severity. Support vector models were performed for each analysis in order to assess multivariate feature contributions to group differences and outcome measures. Based on previous work, we expected to find reduced global efficiency in ADHD relative to TDC. We further hypothesised that prior to treatment, there would be reduced local efficiency in catecholamine-rich regions within cortico-striatal networks in individuals who subsequently respond best to MPH treatment.

## Materials and Methods

### Participant characteristics

DWI data were acquired from 37 children and adolescents with ADHD (8–17 years) and 26 age and gender matched TDC’s. Children with ADHD undertook imaging as part of a baseline research session, prior to commencing 6 weeks of open-label MPH (international Study to Predict Optimised Treatment Response in ADHD; iSPOT-A; Trial registration information: https://clinicaltrials.gov/ct2/show/NCT00863499). A detailed description of diagnosis procedure has been published previously^30^. Briefly, diagnoses were made by referring clinicians based on Diagnostic and Statistical Manual of mental Disorders (4^th^ ed.: DSM-IV) criteria, and confirmed using the Mini International Neuropsychiatric Interview for children and adolescents (MINI-Kid; Sheehan et al.^31^) and the Attention Deficit/Hyperactivity Disorder Rating Scale (ADHD-RS-IV)(administered to the parent/guardian). Typically developing controls were drawn from iSPOT-A and the Limbic Maturational Changes in young Adulthood (LIMCA) study^32^, which used identical experimental procedures on the same scanner. They were screened for Axis 1 mental disorders using the MINI-KID or SPHERE-12^33^. Sample size was based on previous studies investigating whole-brain connectomics using diffusion tensor imaging in child and adolescent ADHD^15,17^.

iSPOT-A received IRB approval from the Human Research Ethics Committee, Western Sydney Local Health District and was conducted according to the principles of the Declaration of Helsinki 2008. All participants (and/or guardian when less than 16 years) provided written informed consent.

### Study treatment

Participants were medication-free at baseline testing and a minimum 5 half-life washout period was applied for those who took medication. ADHD participants were prescribed open-label MPH by their treating paediatrician, which they continued for 6 weeks. Dosage was titrated and optimised as required by their treating paediatrician. During this time, participants refrained from all other ADHD treatments.

### Measures

ADHD symptom severity was assessed using the total score on the parent-completed ADHD-RS-IV. The ADHD-RS-IV assesses symptoms according to the 18 DSM-IV criteria, each symptom rated from 0 to 3. The current analysis uses baseline imaging and clinical symptoms, as well as percent change between baseline (week 0) and Week 6 (post-treatment) clinical symptom scores.

### Imaging acquisition

Data were acquired using an 8-channel head coil on a 3-Tesla GE Sigma HDx scanner (GE Healthcare, Milwaukee, Wisconsin) at the Westmead Hospital, Sydney. Details of the protocol can be found in Supplementary Methods.

### Imaging preprocessing and tractography

DWI data were preprocessed and analysed using the FMRIB Software library (FSL) (v5.0.1) (http://www.fmrib.ox.ac.uk/fsl). Raw DWI data were corrected for head movement and eddy current distortions prior to diffusion tensor models being fitted independently for each voxel within the brain. Data were segmented into 68 cortical regions and seven subcortical structures (amygdala, hippocampus, thalamus, caudate, putamen, palladium and nucleus accumbens) to generate 82 × 82 connectivity matrices. See supplementary methods for details on whole-brain parcellation and tractography matrix generation.

### Graph theory

Graph theoretic analyses were performed on the interregional connectivity matrices (weighted undirected networks) using the Brain Connectivity Toolbox (http://www.brain-connectivity-toolbox.net/)^34^. To avoid biases associated with using a single threshold, we examined topological properties across a range of thresholds (5% < *S* < 30% in steps of 1%) and calculated a single area under the curve (AUC) measure over these thresholds. We calculated the following global network measures: (1) the characteristic path length (the mean number of connections on the shortest path between any two regions in the network); (2) the clustering coefficient, which quantifies the probability that two nodes connected to an index node are also connected with each other and (3) global efficiency, computed as the harmonic mean of the inverse path length. We also examined the local efficiency of individual network regions, which measures the efficiency of the subgraph defined by an index node’s neighbours after removal of that node and putatively indexes fault tolerance. To aid with interpretation, regional results were classified into functional networks defined by Yeo’s 7 network parcellation: visual, somatomotor (SM), dorsal attention (DAN), ventral attention (VAN), limbic, frontoparietal (FP) and default mode network (DMN)^35^, along with SC.

### Statistical analysis

Statistical analysis was designed to address the study aims of (1) Identifying differences between diagnostic groups (ADHD vs TDC), (2) Predicting MPH treatment-related symptom change and (3) Identifying associations with ADHD symptom severity.

*T* tests and ANCOVA’s were used first to test group differences on univariate outcome measures. Associations between graph measures and baseline ADHD symptoms were assessed using correlations, and associations between graph measures and percent change in ADHD symptoms after 6 weeks of MPH treatment were assessed using linear regressions. All analyses were conducted with and without the covariates age, gender, MPH dose (mg/kg), ADHD subtype, duration of previous stimulant treatment and stimulant use in the 6 months prior to study entry. The false discovery rate (FDR) for *t* tests, ANCOVAS, correlations and regression models were corrected for using the Benjamini–Hochberg^36^ method, where *p* values were adjusted to *q* values. The FDR was corrected for each research question (i.e. *n* = 63 when comparing ADHD and TDC, and *n* = 37 for ADHD only) and both *p* and *q* values are provided where appropriate.

Three separate SVMs were completed to test whether the 82 local efficiency measures could be combined to predict diagnostic group, treatment-related symptom change and symptom severity. Model tuning parameters cost (C) and sigma (Σ; in the case of non-linear SVM) were iteratively optimised based on the root mean standard error (RMSE). Models were validated using leave-one-out cross-validation (LOOCV) procedure due to the modest sample size and its superiority in reducing bias and variance of cross-validated accuracy over *k*-fold cross-validation^37^. Model performance for classification prediction was assessed using binomial tests, where each model’s accuracy was compared to its corresponding no information rates (NIR) accuracy. Given the unbalanced nature of some data classes (e.g. ADHD and healthy controls), we also performed permutation testing (1000 times). Model performance for regression prediction was assessed by correlating observed and predicted values. For significant SVM models, we also included covariates to establish whether including covariates improved the model’s performance over local efficiency measures alone. Variable importance in classification SVM models was determined by calculating the area under the receiver operator characteristic (ROC) curve for each variable. Variable importance in regression SVM models was determined by fitting a loess smoother between the outcome and the predictor, then calculating the *r*^2^ statistic for each model against the intercept only null model. All analyses were performed using R 3.6.1^38^. SVM models were generated using the ‘caret'^39^ and ‘kernlab' packages^40^.

Supplementary analyses comparing TDCs vs MPH responders (>25% reduction in symptom severity) and non-responders were also conducted on any significant treatment response univariate results to aid in determining the normative benchmark.

## Results

### Multivariate patterns of local efficiency predict diagnostic group

**Fig. 1:**
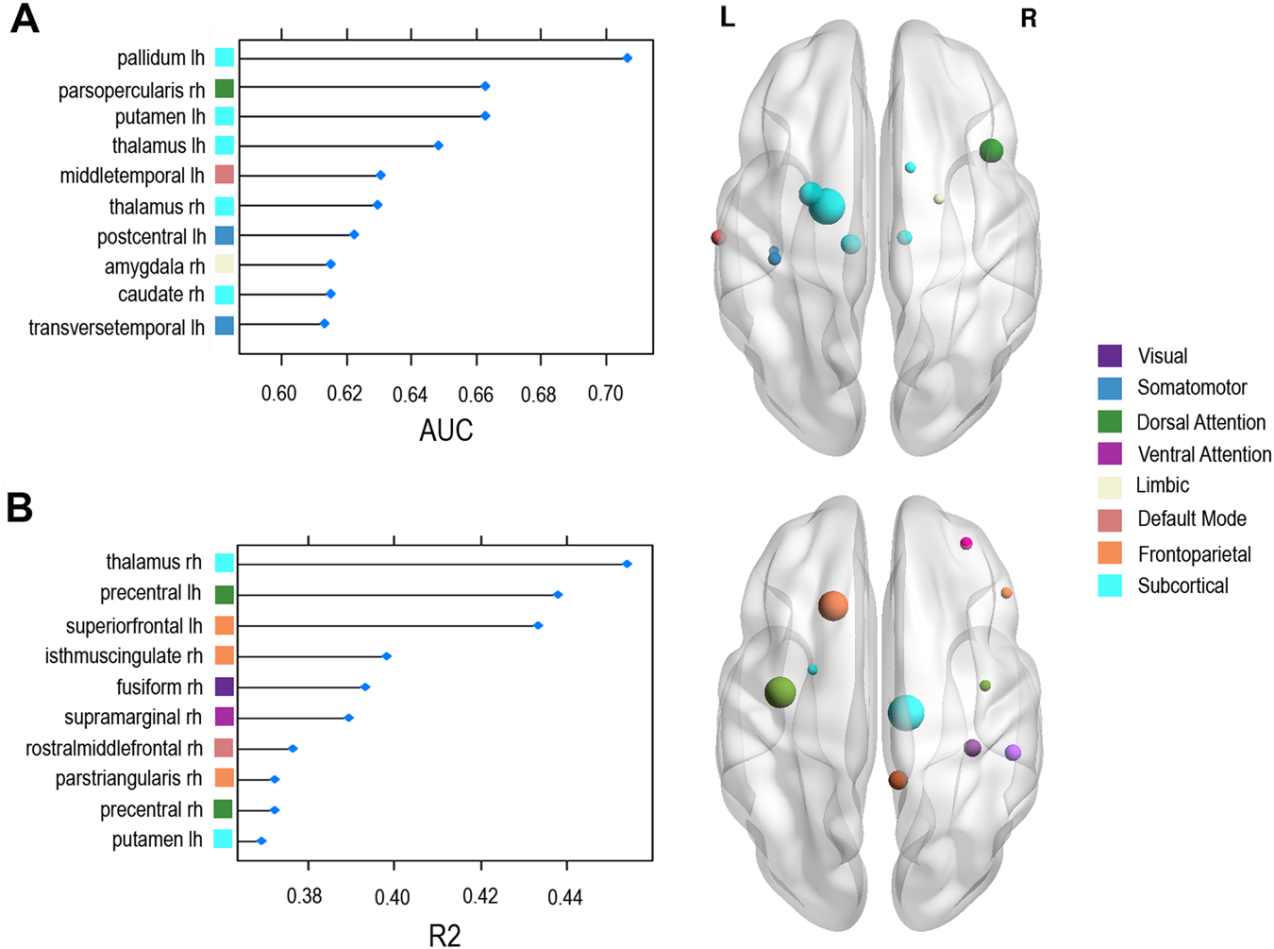
Ranked variable importance for linear SVM models. **A**. Regions contributing most to prediction of diagnostic group (ADHD vs TDC). **B**. Regions contributing most to prediction of change in ADHD symptom severity after 6 weeks of MPH treatment. Representation of location, network and weighting of these regional contributors is shown on the right. For visual clarity, only the top ten most important contributors to the models are shown. Weightings for the remaining contributing brain regions can be found in Supplementary Table 1.

There were no differences between ADHD and TDC groups in global graph measures (global efficiency, *t*(61) = −0.042, *p* = 0.673; characteristic path length, *t*(61) = −0.311, *p* = 0.757; mean clustering coefficient, *t*(61) = 1.035, *p* = 0.258) or univariate measures of nodal local efficiency after correcting for multiple comparisons. However, using linear SVM we found a significant model of combination of local efficiency measures to predict ADHD from TDC with 76.2% accuracy (*C* = 0.218, *p* = 0.003; see Table 1 for cross-validated model details and Fig. 1A for the most important regional contributors). The top ten regions contributing to this model were the left pallidum, putamen and thalamus and right thalamus and caudate (SC), the right pars opercularis (DAN), left mid temporal (DMN), left postcentral and transverse temporal (SM) and right amygdala (limbic), all of which had lower local efficiency in ADHD relative to TDC (see Supplementary Table 1 for a full list of variable contributions to the model). The model was rerun with the covariates age and gender included, however this did not improve the model’s accuracy (see Table 1).

**Table 1:** Accuracy statistics for support vector machine (SVM) classifier models predicting diagnostic group (ADHD vs TDC), using leave-one-out cross-validation.

Performance measures	ADHD vs TDC	
	Local efficiency (82 regions)	Local efficiency + age and gender
Cost	10.9	0.218
Accuracy %	76.2	76.2
CI 95% (lower, upper)	(63.8, 86.0)	(63.8, 86.0)
Accuracy null %	58.7	58.7
Sensitivity	91.9	81.1
Specificity	53.9	69.0
PPV	73.9	79.0
NPV	82.4	72.0
*p* value	0.003	0.003
Permutation *p* value	<0.001	<0.001

Covariates dosage and past stimulant medication excluded as data for these variables does not exist for healthy controls.

*ADHD* attention deficit hyperactivity disorder, *TDC* typically developing children, *CI* confidence interval, *PPV* positive predictive value, *NPV* negative predictive value.

### Global efficiency and local efficiency of the right supramarginal gyrus are predictive of MPH-related change in symptoms

**Fig. 2:**
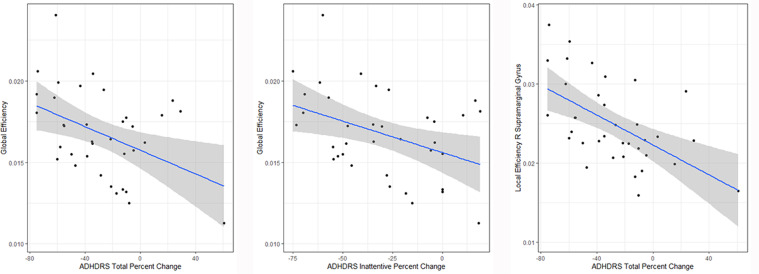
Scatterplots depicting associations between global efficiency and methylphenidate treatment-related change in ADHD total symptom severity and inattentive symptom severity, and change in ADHD-RS total symptom severity and local efficiency of the right supramarginal gyrus.

Higher global efficiency at baseline predicted a greater percent reduction in total ADHD symptom severity (*b* = −5064.1, *t*(34) = −2.76, *p* = 0.009, *q* = 0.027) and a greater percent reduction in inattention symptom severity (*b* = −4234.9, *t*(34) = −2.57, *p* = 0.015, *q* = 0.045) after 6 weeks of MPH treatment. Regionally, higher local efficiency of the right supramarginal gyrus at baseline predicted greater percent reduction in total ADHD symptoms with MPH treatment (*b* = −3319.6, *t*(34) = −3.94, *p* < 0.001, *q* < 0.001) (Fig. 2). These findings all held after adjusting for age, gender, MPH dose, stimulant use in the 6 months prior to study entry, duration of previous stimulant use and ADHD subtype (see supplementary materials). Both global efficiency and local efficiency of the right supramarginal gyrus were lower in MPH non-responders relative to TDCs, while MPH responders and TDCs did not differ (see Supplementary Fig. 1 for these results and Supplementary Table 2 for demographic and clinical characteristics of MPH responders, non-responders and TDCs).

### Multivariate patterns of local efficiency also predict MPH-related change in symptoms

Using linear SVM, multivariate measures of local efficiency predicted change in ADHD symptom severity after 6 weeks of MPH treatment (*r* = 0.37, *p* = 0.029, permutation *p* = 0.036). The top ten regions contributing to this model were the right thalamus (SC), bilateral precentral gyrus (DAN), left superior frontal gyrus, right isthmus cingulate and pars triangularis (FP), right fusiform (visual), right supramarginal gyrus (VAN) and right rostral middle frontal gyrus (DMN), all of which had greater local efficiency associated with greater improvement in symptoms. Adding the covariates age, gender, dosage previous stimulant use, duration of previous stimulant use and ADHD subtype improved the model’s performance (*r* = 0.54, *p* < 0.001, permutation *p* < 0.001). However, these variables did not appear in the top 30 contributing to the model.

### No associations between graph measures and baseline ADHD symptom severity

There were no associations between graph measures and baseline total ADHD symptom severity, inattention symptom severity or hyperactivity symptom severity.

## Discussion

Behavioural disturbances in ADHD are thought to be due to dysfunction of spatially distributed, interconnected neural systems. While there is a fast-growing literature on the impact of functional dysconnectivity in ADHD, far less is known about the structural architecture underpinning these disturbances and how it may contribute to ADHD symptomology and treatment prospects. To address this gap, we used diffusion tractography to examine structural topology in ADHD and TDCs and to explore associations between this white matter organisation and functionally-relevant outcomes such as symptom severity and MPH treatment response. We found that multivariate patterns of reduced local efficiency, predominantly in SC, were able to distinguish the ADHD group from TDCs, while multivariate patterns of increased local efficiency across multiple networks were able to predict better MPH treatment response. Higher global efficiency and higher local efficiency of the right supramarginal gyrus were also associated with an improved prognosis for MPH treatment. Our findings demonstrate that graph measures of structural topology provide valuable diagnostic and prognostic markers of ADHD, which may aid in mechanistic understanding of this complex disorder.

### Multivariate topological measures can predict diagnostic group and MPH treatment prognosis

Forty percent of the regions contributing most to distinguishing ADHD and TDC were SC. Aberrance within the dopamine-rich basal ganglia has long been implicated in the mechanisms of ADHD, and the striatum (caudate and putamen) has been one of the most consistently reported regions of structural and functional difference^41–43^. Altered microstructural properties such as fractional anisotropy have been reported most consistently within the anterior corona radiata and internal capsule in ADHD, which are key tracts surrounding the basal ganglia^8^.While the importance of additional regions such as the parietal lobe and cerebellum have come to light in more recent years^1,2^, the current results demonstrate that dysfunction within the basal ganglia remain a core part of the disorder. Notably, the bilateral caudate, pallidum and thalamus are integral components of cortico-striatal feedback and feedforward loops that are able to modulate activity in a range of cognitive, sensorimotor and limbic circuits^44^. As such, lower local efficiency within these nodes has the capacity to have flow on effects for a broad range of networks and functions. The lack of univariate results suggests that rather than one or two specific regions contributing to group differences, there is a constellation of regions that are wired differently, resulting in a more subtle but diffuse change in information flow.

Meanwhile, the regions contributing most to predicting success of MPH treatment were spread across a range of functional networks, but particularly within the prefrontal cortex. MPH at therapeutic doses preferentially activates catecholamine neurotransmission within the PFC^45^. This could suggest that efficient information flow from and between structures heavily innervated by dopamine and noradrenaline is fundamental to likelihood of good treatment outcomes with MPH. Non-stimulants such as atomoxetine also act on catecholamine systems within similar regions, therefore it would be interesting for future work to examine the specificity of this result to MPH relative to catecholamine therapies more broadly.

### Global efficiency and local efficiency of the right supramarginal gyrus are associated with MPH treatment response

Global efficiency and local efficiency measure how efficiently information is exchanged at the global and local levels, respectively^12^. In our ADHD cohort, higher global efficiency predicted a greater percent reduction in total ADHD symptom severity with MPH treatment. Connectome organisation becomes increasingly integrated across early neurodevelopment, which co-occurs with the synaptic pruning and increased myelination that takes place around puberty^46,47^. This leads to global efficiency increases across childhood and adolescence as communication improves between more disparate subnetworks. Based on this logic, our results suggest that more developmentally advanced global organisation is associated with better MPH treatment response. Notably, this result held after adjusting for age, gender, previous stimulant use and MPH dose.

Higher global efficiency was also predictive of greater treatment-related improvement of inattentive symptoms. This suggests that the significant association between total ADHD symptom severity change and global efficiency is predominantly driven by changes in these symptoms rather than changes in hyperactivity/impulsivity. This could reflect the more diffuse nature of networks supporting attention relative to impulse control.

Regionally, we found that higher local efficiency of the right supramarginal gyrus (VAN) at baseline was associated with better MPH treatment response. The right lateralised VAN is involved in monitoring the environment for behaviourally relevant stimuli^48^, and has previously been reported as having disturbed intrinsic connectivity in ADHD^4^. Notably, the supramarginal gyrus is involved in polymodal sensory integration in the association cortex. The association cortex contains densely connected hubs of the connectome, and has fast rates of myelination and cortical shrinking during adolescence^49^. The rapid neurodevelopment of these hubs during adolescence is argued to be a genetically patterned process of consolidation^49^, which may provide clues to the atypical neurodevelopment seen in ADHD. Based on this one could speculate that possible alterations in gene expression directing neurodevelopment may also be pivotal to ADHD treatment prognosis, via modification of key hub development.

It is important to first note that diffusion tractography cannot determine the precise cortical origins of specific white matter tracts or whether connections are mono- or polysynaptic^50^. However, both primate tracing studies and human in vivo diffusion imaging have demonstrated that the supramarginal gyrus connects with parts of the lateral frontal cortex via the superior longitudinal fasciculus III^51,52^, a tract in which previous studies have reported disturbed microstructure in children with ADHD^53^. Future studies could examine the potential for integrity of this tract to be associated with MPH response in children with ADHD.

Importantly, supplementary analyses revealed that mean local efficiency of the right supramarginal gyrus was lower in MPH non-responders relative to TDCs, whereas MPH responders and TDCs did not differ. This supports the idea that successful treatment with MPH may require intact structural connectivity rather than reflecting a shift from abnormal to normal connectivity. This is a pattern reflected in other disorders and medications; we recently found that depressed patients with greater than normal functional connectivity within key networks were the most likely to benefit from antidepressant treatment^54^.

### No associations between structural topology measures and ADHD symptom severity

We did not observe any significant correlation between symptom severity measures and global or local measures of efficiency. Previous studies found correlations between white matter microstructural properties and ADHD symptoms rather than graph measures^16,17^, which may indicate that tract integrity is associated with current symptomology while structural topology has more bearing on prognosis.

Unlike previous studies, we found lower local efficiency in all regions contributing to diagnostic prediction. Also, while we had anticipated that TDCs would have higher global efficiency relative to ADHD, this was not the case. The supplementary analysis comparing TDCs vs MPH responders and non-responders revealed that this occurred due to heterogeneity within the ADHD group. Mean global efficiency for MPH non-responders and responders groups fell below and above TDCs, respectively.

### Limitations

Our findings should be viewed in light of some limitations. Firstly, the DTI method has difficulty resolving crossing fibre bundles, therefore, providing reduced accuracy of tractography relative to constrained spherical deconvolution-based methods^55^. Fortunately, however, potential errors in tractography are systematically applied, therefore this has minimal impact on our analyses. In addition, the diffusion tractography method utilised counts total streams from the seed, which does not allow for specificity in determining affected white matter tracts or directionality. Secondly, despite all participants undergoing a medication washout period, some participants had a history of stimulant use. This may have already altered structural organisation prior to study entry. While stimulant use in the past 6 months was included as a covariate and did not alter findings, future replication attempts should be completed in a medication naïve sample^56^. Thirdly, given the relatively modest sample size, we were limited to the leave-one-out cross-validation method. While this is superior in reducing bias and variance of accuracy in smaller cohorts, our results should be validated using an independent sample. Finally, we have made speculations on the links with functional measures. A recent multimodal imaging study did find coherence between structural and functional aberrances in a large sample of children with ADHD, as well as associations with clinical features of ADHD^57^. Nonetheless, our results should be validated against functional brain organisation using resting state or functional tasks.

### Summary

Structural connectivity places constraints on which functional interactions occur in the network, particularly during neurodevelopment. Our findings have demonstrated that graph measures of structural topology provide valuable diagnostic and prognostic markers of child and adolescent ADHD. They also highlight that variability in structural topology plays a role in determining the efficacy of MPH response. These new insights contribute to the mechanistic understanding of this complex disorder.

## Supplementary information

Supplementary Methods and Results

Supplementary Figure 1

## Data Availability

R code for statistical analyses is available from the corresponding author upon request.
