# Supplementary material for: Structural brain network topology underpinning ADHD and response to methylphenidate treatment
Source: Transl Psychiatry. 2021 Mar 2;11:150. doi: 10.1038/s41398-021-01278-x (PMC7925571; doi:10.1038/s41398-021-01278-x)
Supplement: Supplementary file 1 — Supplementary Methods and Results [file 41398_2021_1278_MOESM1_ESM.docx]

***Supplementary Methods***

*Imaging Acquisition*

T1-weighted three-dimensional (3-D) spoiled gradient recalled parameters included 180 sagittal 1 mm slices; 1 mm isotropic; 256 × 256 matrix; repetition time = 8.3 msec; echo time = 3.2 msec; flip angle = 11°; and inversion time = 500 msec. Diffusion weighted imaging (DWI) parameters included 70 axial contiguous 2.5 mm slices; 1.72 mm × 1.72 mm resolution; 128 × 128 matrix; repetition time = 17,000 msec; echo time = 95 msec; frequency direction = right/left; 42 diffusion orientations; and b-value = 1250.

*Brain Parcellation and Matrix Generation*

We used Freesurfer (v5.3) (<http://surfer.nmr.mgh.harvard.edu/>) for segmentation of the 3-D T1-weighted structural images, as previously described [31, 32]. In brief, a two-dimensional cortical surface was calculated and automatically divided into 34 gyral-based anatomically labeled areas for each hemisphere using the Desikan-Killiany atlas [33]. An automatic subcortical parcellation was also performed based on probabilistic information of location of subcortical structures automatically estimated from a manually labeled training dataset [34]. Cortical segmentation and anatomical labels were validated by manual inspection

Cortical and subcortical segmentations for each participant were transformed into diffusion space using a 3-D rigid-body six-parameter registration, maximizing [mutual information](https://www.sciencedirect.com/topics/neuroscience/mutual-information) between the non diffusion-weighted image and T1-weighted anatomical data.

We analyzed interregional [white matter](https://www.sciencedirect.com/topics/medicine-and-dentistry/white-matter) connectivity using a multi-fiber diffusion probabilistic model that estimates probability distributions for one or more fiber populations at each brain voxel [30] . These probability distributions guide multiple fiber samples starting from a seed voxel to a specified target region. [Tractography](https://www.sciencedirect.com/topics/medicine-and-dentistry/tractography) was performed using each of the regional labels as seed and the remaining labels as targets. One thousand sample tracts were generated from each voxel within the seed region, and only tracts that reached the target region were retained. Tracts were terminated once they reached a particular target region. For the 41 bilateral brain structures, this resulted in an 82 × 82 interregional connectivity matrix for each participant. Diagonal elements represent self-connections and were excluded from this analysis. The upper half of the diagonal in this matrix contains connections that are mirrored in the lower half. Ideally, these should be symmetric (i.e., the fiber pathway from cortical seed region A to target region B should be equivalent to that from seed region B to target region A). For the purposes of our analysis, the larger of the two values was used to represent the number of probabilistic tracts or fiber pathways that connect the two regions.

To enable comparison of global network properties across participants and groups, we used a sparsity (connection density) threshold (S), which retains S% of the top connections for each participant. This threshold ensured that the number of nodes and connections were matched across participants.

To avoid biases associated with using a single threshold, we examined topological properties across a range of thresholds (5% < *S* < 30% in steps of 1%) and calculated a single area under the curve (AUC) measure over these thresholds. The range of thresholds was selected based on previous work, which has shown that the networks in children and adolescents are small-world within this regime [31]. For each measure, we computed the area under the curve across the full range of sparsity thresholds, and this single non-threshold-biased measure was used for group comparisons.

***Supplementary Results***

*Participant Demographics*

There were no differences between ADHD and TDC groups in age, *t*(61) = 0.09, *p* = 0.93 (ADHD, mean 13.31 ± 2.53; TDC, mean 13.24 ± 2.87) or gender χ^2^(1,63) = 0.417, *p* = 0.52. In the ADHD group, the mean ADHD-RS total severity score was 35.38 ± 7.41 (out of a possible 54). Seventeen were diagnosed with inattentive subtype, while 20 had combined subtype. Nineteen of the ADHD group (51%) had used stimulants in the 6 months prior to study entry. The mean study MPH dose was 35.99mg ± 17.48, or 0.66mg/kg ± 0.04.

Table 1. Ranked variable importance in regression SVM predicting diagnostic group

| **Variables** | **AUC** | **Importance** |  | **Variables** | **AUC** | **Importance** |
| --- | --- | --- | --- | --- | --- | --- |
| pallidum lh | 0.71 | 100.00 |  | temporalpole lh | 0.56 | 29.29 |
| parsopercularis rh | 0.66 | 78.79 |  | amygdala lh | 0.56 | 28.79 |
| putamen lh | 0.66 | 78.79 |  | superiorparietal rh | 0.56 | 28.28 |
| thalamus lh | 0.65 | 71.72 |  | isthmuscingulate rh | 0.56 | 26.77 |
| middletemporal lh | 0.63 | 63.13 |  | posteriorcingulate rh | 0.55 | 25.76 |
| thalamus rh | 0.63 | 62.63 |  | lateraloccipital lh | 0.55 | 25.25 |
| postcentral lh | 0.62 | 59.09 |  | parahippocampal lh | 0.55 | 21.72 |
| caudate rh | 0.62 | 55.56 |  | medialorbitofrontal lh | 0.54 | 21.21 |
| amygdala rh | 0.62 | 55.56 |  | middletemporal rh | 0.54 | 19.19 |
| transversetemporal lh | 0.61 | 54.55 |  | cuneus rh | 0.54 | 18.69 |
| precentral lh | 0.61 | 53.03 |  | lingual rh | 0.54 | 18.69 |
| entorhinal lh | 0.61 | 52.53 |  | Gender | 0.54 | 17.93 |
| pallidum rh | 0.61 | 52.53 |  | inferiorparietal rh | 0.54 | 17.68 |
| frontalpole lh | 0.61 | 51.01 |  | insula rh | 0.54 | 17.68 |
| entorhinal rh | 0.61 | 51.01 |  | lingual lh | 0.54 | 17.17 |
| caudalanteriorcingulate rh | 0.60 | 50.51 |  | superiorfrontal lh | 0.53 | 16.16 |
| caudate lh | 0.60 | 48.99 |  | medialorbitofrontal rh | 0.53 | 16.16 |
| supramarginal rh | 0.60 | 45.96 |  | pericalcarine rh | 0.53 | 15.15 |
| inferiortemporal lh | 0.59 | 45.45 |  | bankssts lh | 0.53 | 13.64 |
| superiortemporal lh | 0.59 | 45.45 |  | rostralanteriorcingulate rh | 0.53 | 13.64 |
| putamen rh | 0.59 | 44.95 |  | cuneus lh | 0.52 | 11.62 |
| parsorbitalis lh | 0.59 | 42.93 |  | frontalpole rh | 0.52 | 11.62 |
| insula lh | 0.59 | 41.92 |  | precuneus rh | 0.52 | 10.61 |
| accumbens lh | 0.59 | 40.91 |  | superiorfrontal rh | 0.52 | 10.61 |
| rostralmiddlefrontal lh | 0.58 | 40.40 |  | superiortemporal rh | 0.52 | 9.60 |
| postcentral rh | 0.58 | 40.40 |  | supramarginal lh | 0.52 | 8.59 |
| hippocampus rh | 0.58 | 39.39 |  | precuneus lh | 0.52 | 7.58 |
| precentral rh | 0.58 | 38.89 |  | rostralanteriorcingulate lh | 0.51 | 6.06 |
| temporalpole rh | 0.58 | 38.89 |  | parsorbitalis rh | 0.51 | 6.06 |
| caudalmiddlefrontal lh | 0.58 | 37.88 |  | pericalcarine lh | 0.51 | 5.56 |
| parsopercularis lh | 0.58 | 37.37 |  | bankssts rh | 0.51 | 5.05 |
| hippocampus lh | 0.58 | 36.36 |  | transversetemporal rh | 0.51 | 5.05 |
| parstriangularis lh | 0.57 | 35.86 |  | caudalanteriorcingulate lh | 0.51 | 4.55 |
| inferiortemporal rh | 0.57 | 35.86 |  | inferiorparietal lh | 0.51 | 4.55 |
| fusiform lh | 0.57 | 35.35 |  | accumbens rh | 0.51 | 4.55 |
| paracentral lh | 0.57 | 35.35 |  | superiorparietal lh | 0.51 | 4.04 |
| parstriangularis rh | 0.57 | 35.35 |  | parahippocampal rh | 0.51 | 3.54 |
| caudalmiddlefrontal rh | 0.57 | 32.83 |  | posteriorcingulate lh | 0.51 | 2.02 |
| rostralmiddlefrontal rh | 0.57 | 32.32 |  | Age | 0.51 | 2.02 |
| isthmuscingulate lh | 0.56 | 30.81 |  | lateralorbitofrontal rh | 0.50 | 1.52 |
| fusiform rh | 0.56 | 29.80 |  | lateralorbitofrontal lh | 0.50 | 0.00 |
| paracentral rh | 0.56 | 29.80 |  | lateraloccipital rh | 0.50 | 0.00 |

AUC, area under the curve; rh, right hand side; lh, left hand side

**Prediction of MPH response after adjusting for covariates**

All results remained significant after adjusting for covariates (age, gender, previous stimulant medication use, dosage at week 8, duration of previous stimulant use, subtype): Global efficiency and percent reduction in total ADHD symptom severity (*b* = -7087.4, *t*(27) = -3.75, *p* = 0.001), global efficiency and percent reduction in inattention ADHD symptom severity (*b* = -5646.9, *t*(27) = -3.21, *p* = 0.004), and right supramarginal gyrus and percent reduction in total ADHD symptom severity (*b* = -4086.2, *t*(27) = -4.82, *p* < 0.001).

**Categorically-defined MPH response – supporting analyses**

20 participants experienced a ≥25% reduction in ADHD-RS scores (M -50.66, SD 15.28), while 16 did not (M -0.20, SD 22.10). Table 1 shows the demographic and clinical characteristics of MPH responders and non-responders. There were no significant differences in age, gender, baseline ADHD-RS severity scores, week 8 dose, or stimulant use in past 6 months between MPH responders and non-responders.

*Table 2. Demographic and clinical characteristics of MPH responders and non-responders*

|  | **Responders (n=20)** | **Non-responders (n=16)** | **TD controls (n=24)** | **t/χ^2^(p value)** |
| --- | --- | --- | --- | --- |
| Age (years) | 13.1 (2.5) | 13.5 (2.7) | 13.3 (2.7) | 0.77 (0.44) |
| Male, n(%) | 14 (70) | 12 (75) | 16 (67) | 0.32 (0.85) |
| *Baseline ADHD-RS severity* |  |  |  |  |
| Total /54 | 36.9 (7.1) | 33.9 (7.8) |  | -1.17 (0.25) |
| Hyperactivity-Impulsivity /27 | 15.1 (6.3) | 12.7 (6.6) |  | -1.11 (0.27) |
| Inattention /27 | 21.8 (3.5) | 21.3 (3.4) |  | -0.43 (0.67) |
| MPH week 8 dose (mg) | 37.2 (21.6) | 34.5 (11.4) |  | -0.45 (0.66) |
| Stimulant use past 6 months, n (%) | 9 (45) | 10 (62.5) |  | 1.09 (0.30) |

**Global Efficiency**

Global efficiency was lower in MPH non-responders (*M* = 0.016, *SD* = 0.002) compared to responders (*M* = 0.018, *SD* = 0.002, *t*(34) = -2.794, *p* =0.008, *q* = 0.024). TDC (*M* = 0.017, *SD* = 0.002) did not differ from responders, *t(46) =* 0.86*, p=*0.393*, q=*0.59 or non-responders, *t(42) =* -2.02*, p=*0.05*, q=*0.149*.*

**Local Efficiency of Right Supramarginal Gyrus**

Local efficiency was lower in MPH non-responders (*M* = 0.022, *SD* = 0.004) compared to responders (*M* = 0.027, *SD* = 0.005), *t*(34) = -3.47, *p*=0.001, *q* = 0.017, and TDCs (*M* = 0.026, *SD* = 0.006),*t*(40) = -2.61, *p*=0.013, *q*=0.017. There were no significant differences between responders and TDCs, *t*(44) = 0.71, *p*=0.482.

*Supplementary Figure 1. Comparison of mean global efficiency and local efficiency of the right Supramarginal gyrus in methylphenidate (MPH) non-responders, responders, and typically developing controls (TDC). Error bars reflect standard error from the mean (SEM). Asterisk denotes statistical significance at p<0.05, corrected for multiple comparisons.*
