# Supplementary figures and images for: Structural brain network topology underpinning ADHD and response to methylphenidate treatment
Source: Transl Psychiatry. 2021 Mar 2;11:150. doi: 10.1038/s41398-021-01278-x (PMC7925571; doi:10.1038/s41398-021-01278-x)

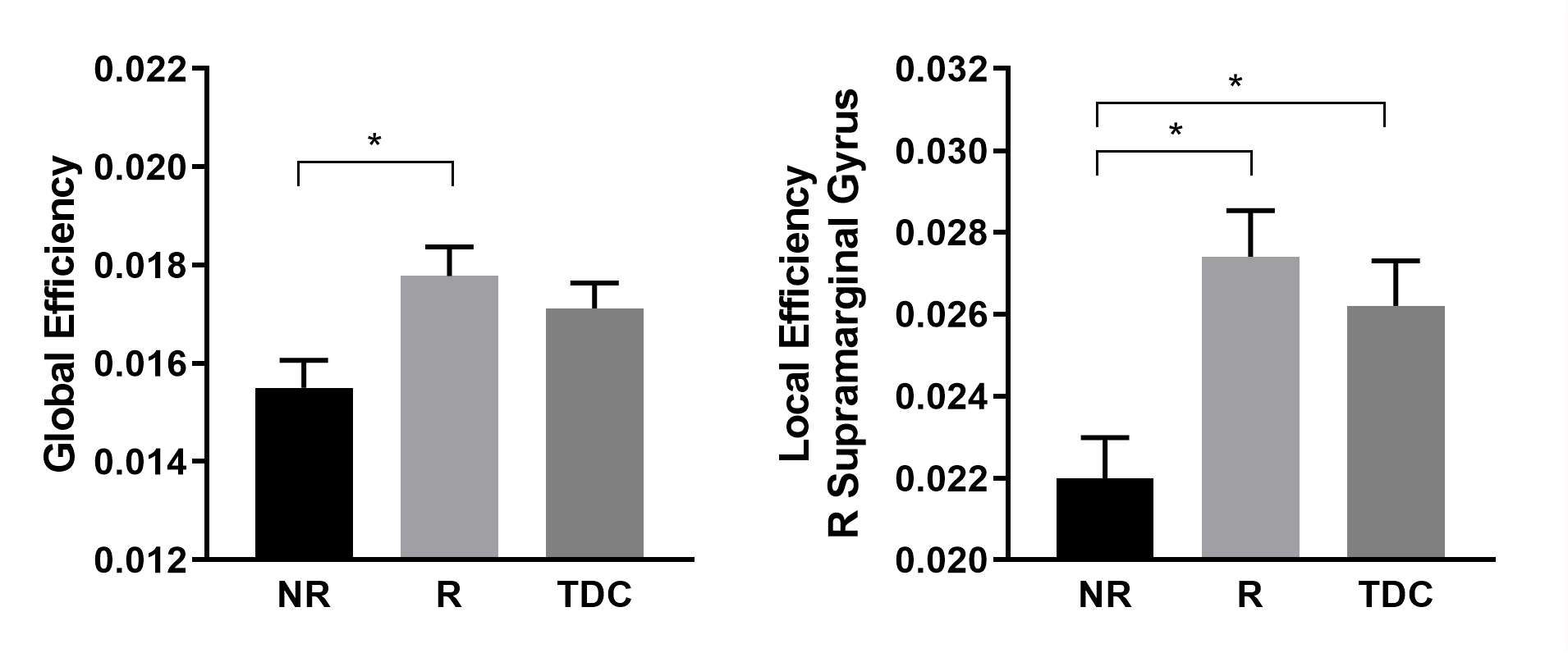

Supplement: Supplementary file 2 — Supplementary Figure 1 [file 41398_2021_1278_MOESM2_ESM.tif]
